# Avoiding monocular artifacts in clinical stereotests presented on column-interleaved digital stereoscopic displays

**DOI:** 10.1167/16.14.13

**Published:** 2016-11-16

**Authors:** Ignacio Serrano-Pedraza, Kathleen Vancleef, Jenny C. A. Read

**Affiliations:** iserrano@ucm.es https://www.researchgate.net/profile/Ignacio_Serrano-Pedraza; Kathleen.Vancleef@newcastle.ac.uk; jenny.read@newcastle.ac.uk http://www.jennyreadresearch.com/; Faculty of Psychology, Universidad Complutense de Madrid, Madrid, Spain; Institute of Neuroscience, Newcastle University, Newcastle upon Tyne, UK; Institute of Neuroscience, Newcastle University, Newcastle upon Tyne, UK; Institute of Neuroscience, Newcastle University, Newcastle upon Tyne, UK

**Keywords:** *monocular artifacts*, *column-interleaved displays*, *stereopsis*, *random-dot stereograms*, *stereoacuity*

## Abstract

New forms of stereoscopic 3-D technology offer vision scientists new opportunities for research, but also come with distinct problems. Here we consider autostereo displays where the two eyes' images are spatially interleaved in alternating columns of pixels and no glasses or special optics are required. Column-interleaved displays produce an excellent stereoscopic effect, but subtle changes in the angle of view can increase cross talk or even interchange the left and right eyes' images. This creates several challenges to the presentation of cyclopean stereograms (containing structure which is only detectable by binocular vision). We discuss the potential artifacts, including one that is unique to column-interleaved displays, whereby scene elements such as dots in a random-dot stereogram appear wider or narrower depending on the sign of their disparity. We derive an algorithm for creating stimuli which are free from this artifact. We show that this and other artifacts can be avoided by (a) using a task which is robust to disparity-sign inversion—for example, a disparity-detection rather than discrimination task—(b) using our proposed algorithm to ensure that parallax is applied symmetrically on the column-interleaved display, and (c) using a dynamic stimulus to avoid monocular artifacts from motion parallax. In order to test our recommendations, we performed two experiments using a stereoacuity task implemented with a parallax-barrier tablet. Our results confirm that these recommendations eliminate the artifacts. We believe that these recommendations will be useful to vision scientists interested in running stereo psychophysics experiments using parallax-barrier and other column-interleaved digital displays.

## Introduction

Technical advances are offering vision scientists new ways of displaying stereo images. One example is the development of autostereoscopic 3-D displays, which do not require the use of eyeglasses or mirrors to view them. The two most common multiplexing techniques used in autostereo displays are the parallax barrier (using opaque layers) and the microlens technique (using cylindrical lenslets; Dodgson, 2005; Holliman, Dodgson, Favalora, & Pockett, 2011; Konrad & Halle, 2007; Peterka et al., 2008; Sexton & Surman, 1999). In this work we will focus on the parallax-barrier technique, which produces a column-interleaved stereo display. The parallax barrier is in fact one of the oldest autostereoscopic techniques (Sexton & Surman, 1999): In 1838, Wheatstone proposed a simple version of this technique (see his figure 6) in order to help fuse two disparate images when the naked eyes are used.

**Figure 1:**
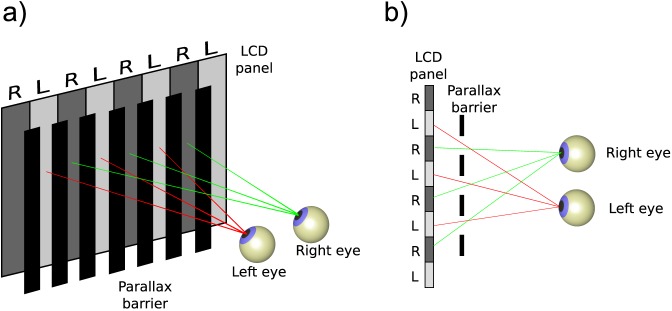
Basic idea behind the parallax-barrier technology. (a) A perspective example. (b) A top view.

As generally implemented today, digital parallax-barrier stereoscopic displays consist of two LCD panels overlaid on one another with a precise geometric relationship (Konrad & Halle, 2007). The back panel is used as normal to display the images, and the front is transparent when the device is used in 2-D mode. When the 3-D display is activated, the front LCD panel generates opaque vertical grid lines (Figure 1). When the viewer is in the right position, odd columns of pixels are visible only to one eye, while even columns are visible only to the other. Thus this technique allows two disparate images to be presented in column-interleaved format on a single screen, one image to each eye, so they can be fused without using 3-D glasses.

One limitation of these parallax-barrier displays is that the spatial resolution of the image in the horizontal dimension is halved. A further limitation is that the occlusion geometry only holds over a limited range of viewing distances and only if the display is frontoparallel, so that the interocular axis is parallel to the screen plane. If the display is rotated even slightly away from frontoparallel (e.g., viewed from the side), both images will become visible to both eyes. This can also occur if the observer views the display from the wrong distance, or with more extreme rotations about the interocular axis (e.g., if the display is viewed from above or below; see Figure 11A). With more extreme rotations away from frontoparallel (more oblique viewing, e.g., if the display is viewed from the left or right side), the images can become inverted so that the image intended for the right eye will be visible only to the left, and vice versa.

In this article, we consider the use of column-interleaved autostereo digital technology for clinical tests of stereoacuity, or stereotests. This technology has three main advantages that make it particularly attractive for clinical applications: (a) It does not require special glasses or other optics, which is especially useful with children; (b) it offers very low interocular cross talk when correctly positioned; and (c) it is available on handheld mobile devices, making it portable and convenient.

**Figure 2:**
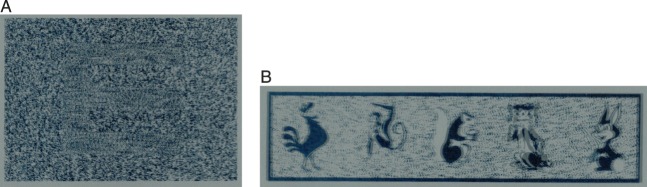
Possible cross-talk artifacts in the Randot stereotest. (A) Random-dot component, in which the task is to recognize the disparate symbol (the letter *E*). (B) Animals component, in which the task is to identify the nearest animal (the cat, fourth from the left). If the viewer removes his or her 3-D glasses or tilts his or her head 45°, so that both eyes view the merged binocular image instead of each eye seeing its intended image, the correct answer is visible monocularly in both cases.

**Figure 3:**
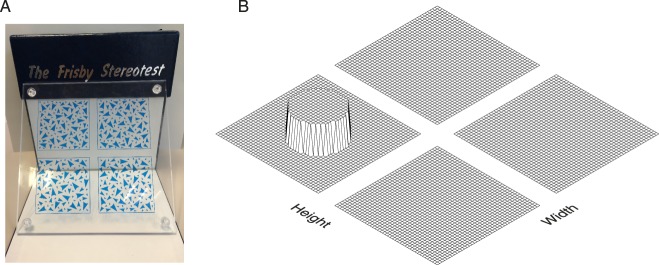
(A) Frisby stereotest. (B) Schematic idea of a disparity-detection test. Four textured patches are presented, all at the same background parallax except one, which contains a target at a different parallax. The task is to identify the patch containing the target.

The fundamental requirement of a stereoacuity test is that it must not have monocular cues (e.g., occlusion, texture gradient, relative size, motion parallax) that enable observers to perform correctly without using their stereovision. Many current clinical stereotests, such as the Randot (Figure 2), Frisby (Figure 3), TNO, Lang, and Random-Dot E tests, try to avoid the presence of monocular depth cues by using random-element stereograms (Julesz, 1960). The subject has to detect a target which—ideally—is perfectly camouflaged in the monocular images and can be detected only by stereopsis (in Julesz's terminology, the target is cyclopean). In all of these tests, monocular artifacts are a potential issue if care is not taken in the administration of the test.

Most current stereotests use 3-D glasses, either polarized (Randot, Random-Dot E) or anaglyph (TNO), to separate the images presented to each eye. The use of 3-D glasses often allows significant cross talk between the two eyes—that is, some fraction of the image that is presented to one eye also passes through the filter that covers the other eye. Cross talk is undesirable because it reduces stereoacuity and depth perception (Cormack, Stevenson, & Landers, 1997; Schor & Heckmann, 1989). Potentially, cross talk can also “de-camouflage” a cyclopean object, enabling a stereoblind user to detect a target which was intended to be visible only to stereopsis. For example, in the Randot stereotest, subjects are asked to identify cyclopean shapes in a random-dot pattern. When the test is viewed without glasses, the disparate dots appear twice, meaning that the shapes are clearly visible monocularly (Figure 2A). The same problem also applies even with glasses if the subject tilts his or her head. The Randot stereotest uses linear polarization to separate left and right images, so a head tilt of 45° results in 100% cross talk.

The Lang and Frisby stereotests both avoid the need for 3-D glasses. The Lang stereotest uses column-interleaved autostereo lenticular technology, in which lenses are used to direct each image to the appropriate eye. If the subject is allowed to tilt the test card back and forth, he or she can identify the disparate shape from the monocular motion (i.e., motion parallax) as the left and right images alternate. The Frisby stereotest (Figure 3) uses physical depth; it consists of three transparent plates of varying thickness, each printed with a pattern. The subject is required to find the part of the pattern which is printed on the opposite surface of the plate. This is intended to be done by detecting the disparity between the front and back surfaces of the plate. Since the Frisby stereotest uses physical depth, there is no cross talk, but motion parallax (e.g., lateral movements of the head) can again enable subjects to identify the target without requiring stereopsis.

Thus, in all current stereotests, monocular artifacts are a potential issue to a greater or lesser extent. Many test protocols advise repeating the stereotest with one eye occluded. If stereoacuity is not impaired, the tester concludes that the previous value was due to monocular artifacts.

Many of these problems apply also to column-interleaved digital displays. These displays also have significant cross talk if the viewer views the display at an angle. They also allow monocular motion artifacts if the viewer moves relative to the display. In addition, we will report a more subtle artifact that is specific to column-interleaved stereo displays: When random-element cyclopean content generated for a non-column-interleaved display is displayed in column-interleaved format, the elements appear wider or narrower on the screen depending on the sign of their disparity (near or far relative to the screen). When combined with cross-talk artifacts, this could also allow observers to detect the stimulus using monocular cues. We will show examples of this artifact and a procedure to get rid of it.

However, the critical advantage of digital displays over the paper and plastic used for current clinical stereotests is that it is possible to design a stereotest to be robust to all these artifacts. We will explain how to achieve this, and will show data indicating that after our strategies are applied, viewers cannot achieve artificially good results through the use of monocular cues. With these strategies, digital column-interleaved 3-D displays are far more robust to monocular-cue artifacts than are current clinical stereotests.

## Problems presenting cyclopean stimuli in column-interleaved display

We consider how to present a cyclopean stereoacuity task on a column-interleaved autostereo display such as the NEO3DO parallax-barrier display (see the Methods subsection) in such a way that accurate performance requires the use of stereopsis. To this end, we need to eliminate monocular artifacts which would enable the task to be performed by a stereoblind or monocular observer.

### Definitions

We use the term *parallax* to refer to the shift on the screen between left and right half-images, reserving the term *disparity* for the angular quantity measured at the retina. We adopt the convention that more negative parallaxes depict objects further in front of the screen plane. We define a stereogram as consisting of a pair of *half-images*, one presented to the left eye and the other to the right. Each half-image is of course made up of pixels, and we shall refer to these as *half-image pixels* or *H-pixels*. We shall use the term *interleaved pixels* or *I-pixels* to refer to the physical pixels on a column-interleaved display.

### Problem 1: Parallax inversion

As discussed already, if a parallax-barrier display is viewed from a sufficiently oblique angle—for example, if it is held in a slanted position about a vertical axis—the image intended for the left eye may be visible to the right, and vice versa. The parallax is therefore inverted. For this reason, it is hazardous to use a disparity-discrimination task on such a display (i.e., to ask the viewer which of two objects appears closer) unless the relative positions of the viewer and display are fixed. If the viewer is allowed to hold the device, there is a chance he or she may hold it at such an angle as to invert the parallax, and so responses would be systematically incorrect.

### Solution 1: Use a task that is robust to parallax inversion

This problem is avoided by using a detection task—that is, requiring people to detect the presence of a disparity change rather than discriminate its sign. Most clinical stereotests are already robust to disparity inversion, even those that require glasses and thus are not vulnerable to inversion as a practical problem. The Lang stereotest and the random-dot component of the Randot ask the user to discriminate the shape of a disparity-defined outline. The Frisby test and the circles and animals components of the Randot use a detection or odd-one-out task. For example, the Frisby stereotest (Figure 3A) contains four patches, three of which have uniform disparity while one contains a disparate target (Figure 3B). The Frisby stereotest is similar to a parallax-barrier display in that it is easy to invert depth by turning the plate around, but this does not affect the identity of the target patch. Accordingly, the test protocol does not require the clinician to present the plate in a particular orientation, but allows them to ask the patient to “find the hidden target” or “find the hidden hole.” In our experiments, we will present an analogous task on a parallax-barrier display using a random-dot pattern. That is, we will present distractor patches of random-dot patterns all with the same background parallax and one patch which contains a region with the target parallax; the task is to locate the target.

### Problem 2: Cross talk can de-camouflage a cyclopean target

**Figure 4:**
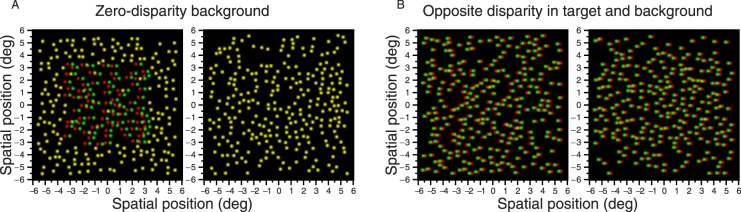
Example stimuli from a two-alternative forced-choice disparity-detection task, presented in anaglyph. Each panel depicts two patches of random dots, the right-hand one with uniform disparity and the left-hand with a disparate, near target. The task is to identify which patch contains the target (the red filter should be placed in front of the right eye). (A) Disparate target on zero-disparity background. If the viewer removes 3-D glasses, so that both eyes view the merged binocular image instead of each eye seeing its intended image, the target is immediately visible, since the dots are doubled. (B) Symmetrical parallax: Target and background have equal but opposite disparity. The target is now no longer obvious in the merged binocular image, although in the case of anaglyph, it can be revealed by a careful comparison of the relative locations of green and red dots. This is a problem affecting displays where the left and right images can be not only perceived together but distinguished.

The value of using a random-dot stereogram is that the target is perfectly camouflaged when viewed monocularly: It is cyclopean, only detectable by stereopsis. However, interocular cross talk can cause a target that is intended to be cyclopean to become visible monocularly. We saw in Figure 2 how this affects the Randot stereotest. Figure 4 shows the effect for a random-dot stereogram like those used in our experiments, presented for viewing with anaglyph 3-D glasses (red lens over the right eye, green over the left eye). Figure 4A and B depict two patches of random dots each, one of which has uniform disparity and the other of which contains a near-disparity square target floating in front of the background. The task is to detect the patch containing the target, which is the left patch in both examples. Viewing the figure without 3-D glasses, we see the merged binocular image (i.e., with total cross talk: Both eyes see both half-images). In the merged binocular image, the target is immediately visible in Figure 4A because the dots composing it appear double. We will call this phenomenon *cross-talk de-camouflage*. As already discussed, it affects nearly all clinical stereoacuity tests to some degree (Figure 3). Although cross-talk de-camouflage is not specific to column-interleaved displays, the problem is particularly acute for these and other autostereo displays, because even small head movements allow the viewer to catch a glimpse of the merged binocular image.

### Solution 2: Apply parallax symmetrically to avoid cross-talk de-camouflage

The problem of cross-talk de-camouflage is greatly reduced by applying parallax symmetrically—that is, giving target and background equal and opposite parallax. Figure 4B shows a random-dot stereogram where the relative parallax between target and background is the same as in Figure 4A, but now the parallax has been applied symmetrically. In this anaglyph image, it is still possible to identify the target by careful inspection of the colored dots: The left dot of the pair of dots that are part of the target has red color, while for background dots it is the opposite. In a display using more modern technology to separate the images—for example, circular or linear polarization—this cue essentially disappears. Applying parallax symmetrically can therefore restore the camouflage.

In order to apply this technique correctly, the target and background should have the same magnitude of parallax. In the Randot stereotest, the “background” parts of the stimulus (e.g., the frame, speckled background, and distractor objects in the animals component) have the opposite sign of disparity to the foreground, but they still have different magnitudes (Figure 3). This means that the target object can still be identified when the test is viewed without glasses or with the head rotated. This could have been avoided if the parallax had been of equal magnitude in the target and background.

### Problem 3: Avoiding cross-talk de-camouflage on column-interleaved displays

**Figure 5:**
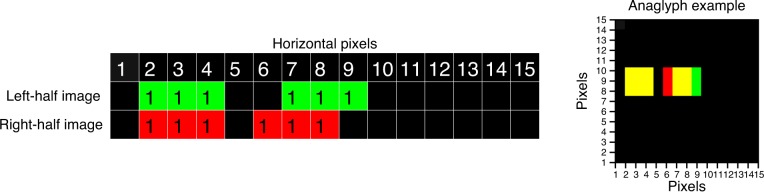
Schematic diagram of a stereogram (on a non-column-interleaved display) in which each half-image contains two dots, each 3 pixels wide. Pixels covered by the dots are labeled 1 (for comparison with later cases when we consider antialiasing). The pixels are also color-coded to make it easier to see which eye they are intended for. The first dot has a parallax of 0 horizontal pixels (same position in both half-images), while the second dot has a parallax of 1 pixel. The image on the right presents the stereogram in an anaglyph version (the red filter should be placed in front of the right eye).

There is a particular subtlety in applying parallax symmetrically on column-interleaved displays. The definition of parallax is complex on such displays because even a notionally zero-disparity image necessarily has one pixel of parallax on the display. For example, consider the stereogram shown in Figure 5. In Figure 5A, the left and right half-images are shown offset vertically. This might literally be the case on a row-interleaved stereo display (e.g., a patterned-retarder passive 3-D monitor), or the images might be optically superimposed (e.g., in a mirror haploscope or in a two-projector stereo system); as far as the horizontal parallax in the image is concerned, all these systems are the same. This simple stereogram contains two dots, each 3 pixels wide. The first dot is at the same position, Pixels 2–4, in the left and right half-images—that is, it has zero parallax. The second dot has a parallax of 1 pixel—that is, the dot is shifted rightward by 1 pixel in the left eye's half-image relative to the right eye's half-image.

**Figure 6:**
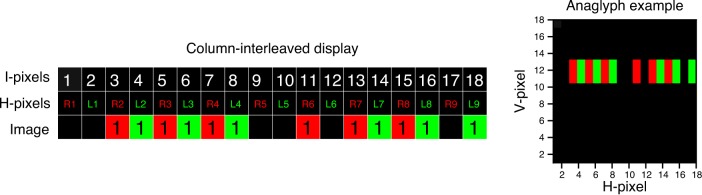
Stereogram of Figure 5, drawn on a column-interleaved display. The image on the right presents the dots in an anaglyph version (the red filter should be placed in front of the right eye). Note than in a real display the columns are thinner, so the black lines in the fused dots are almost invisible.

Now imagine trying to depict this stereogram on a column-interleaved display. In a column-interleaved display, the number of pixels available for each half-image horizontally is half the number of physical pixels across the screen. We will refer to these physical pixels as interleaved or I-pixels, and will use the term H-pixels to refer to pixels of the left and right half-images. Figure 6 shows how the stereogram of Figure 5 would be drawn on a column-interleaved display where the right half-image is drawn on the odd I-pixels and the left half-image is drawn on the even I-pixels.

**Figure 7:**
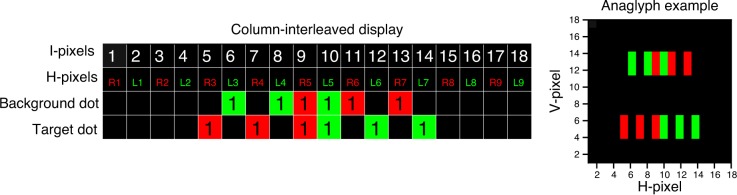
Cross-talk de-camouflage artifact: Example of target and background dots which can be distinguished monocularly. In each image, the dots are 3 H-pixels wide, as in Figure 3, but the target dot extends across more pixels on the interleaved display (spanning I-pixels 5 through 14, as opposed to 6 through 13 for the background dot). The image on the right presents the dots in an anaglyph version (the red filter should be placed in front of the right eye).

As can be seen in Figure 6, column interleaving affects horizontal parallax. In the original half-images (Figure 5), the first dot has zero parallax. However, when these half-images are presented on a column-interleaved display (Figure 6), this dot has a parallax of 1 I-pixel, since the left half-image of the dot appears 1 I-pixel to the right of the right half-image. This means that the dot appears in front of the plane of the physical screen. The second dot has a parallax of 1 H-pixel as drawn in the original half-images; however, it now has a parallax of 3 I-pixels on the column-interleaved display. We can distinguish H-parallax *D*_H_, the parallax of the half-images in H-pixels, from I-parallax *D*_I_, the parallax on the column-interleaved display in I-pixels. These are related by

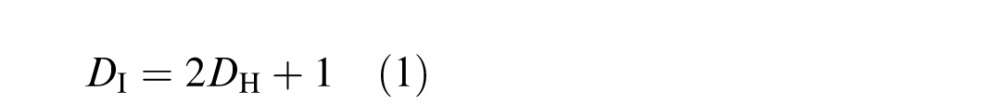
where *D* is the distance in pixels between left and right images. This distinction is important because, to avoid cross-talk de-camouflage on a column-interleaved display, it is the I-parallax, *not* the H-parallax, that must be applied symmetrically. As a counterexample, Figure 7 shows an example where H-parallax is applied symmetrically. Here the two rows represent a background dot and a target dot, with H-parallax *D*_H_ = ±2 H-pixels. For example, the background dot begins at H-pixel L3 in the left eye and R5 in the right eye, for an H-parallax of *D*_HB_ = −2 H-pixels, whereas the target dot begins at L5 and R3, for an H-parallax of *D*_HT_ = −2 H-pixels. Both dots are the same size, 3 H-pixels, in each half-image individually, but in the merged binocular image the background dot is smaller. It spans only 8 I-pixels, from 6 to 13, compared to 10 I-pixels for the target dot. Of course, this is only an issue when cross talk makes the merged binocular image visible.

**Figure 8:**
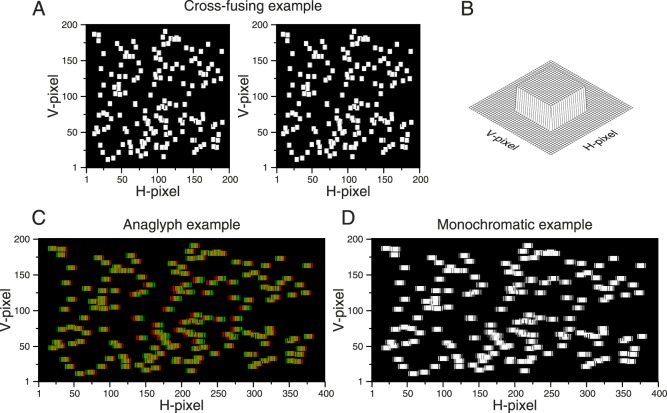
Example of a random-dot stereogram presented on a column-interleaved display showing the artifact. (A) Left and right images of the stereogram for cross-fusing. Background dots have a parallax of −2 H-pixels and the target dots have a parallax of 2 H-pixels. Note that the dot position was not restricted to integer disparities, and antialiasing was used to depict noninteger positions. In this example we do not need antialiasing, but in general we may wish to display subpixel disparities. (B) Sketched version of the stereogram representing the 3-D percept. (C) Anaglyph version. Left and right images are drawn interleaved (the red filter should be placed in front of the right eye). Note that the target dots are wider than the background dots. (D) Monochromatic version of panel C where the target can be discriminated from the background. (Due to the column interleaving, the dots in panels C and D are now rectangular, but the aspect ratio could be changed easily by increasing the height of the dot to produce a square dot on the screen.)

Figure 8 shows what happens when an entire random-dot pattern made in this way is displayed on a column-interleaved parallax-barrier display. Under close examination, the target can be detected in the merged binocular image (Figure 8C, D) despite the lack of stereo cues. This could potentially enable a stereoblind observer to perform the task.

This artifact occurs because although the H-parallax in the half-images is symmetrical, the I-parallax on the column-interleaved display is not. In Figure 7, the background dot (top row) has a disparity I-parallax of *D*_IB_ = −3 I-pixels in the interleaved image (since it starts at I-pixel 6 in the left eye and I-pixel 9 in the right eye). The target dot (bottom row in Figure 7) has *D*_HT_ = 2 H-pixels in the half-images (since it starts at H-pixel 5 in the left eye and H-pixel 3 in the right eye) but *D*_IT_ = 5 I-pixels in the interleaved image (it starts at I-pixel 5 in the right eye and I-pixel 10 in the left eye). Thus, although the H-parallax is symmetrical, differing only in sign between target and background dots, the I-parallax is not, differing in both sign and magnitude. As a result, the target and background dots occupy different extents in the interleaved image: 10 and 8 I-pixels, respectively. Cross talk converts this difference into a monocular cue.

### Solution 3: Apply I-parallax symmetrically

**Figure 9:**
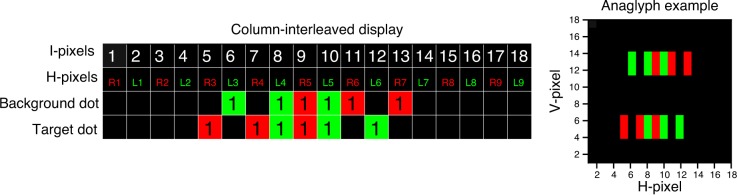
Example corrected for cross-talk de-camouflage artifact. As in Figure 7, except the target dot has been shifted −1 H-pixel in the left eye (a negative shift meaning leftward). Now, background and target dots have the same width (the target dot spans I-pixels 5 through 12 and the background dot spans 6 through 13).

To avoid this kind of monocular artifact in column-interleaved displays, we need to apply I-parallax symmetrically. As we saw in Equation 1, the I-parallax *D*_I_ of physical pixels in the column-interleaved display is related to the H-parallax *D*_H_ of pixels in the half-images by *D*_I_ = 2*D*_H_ + 1. Therefore, to make the I-parallax of the interleaved images symmetrical between target and background, *D*_IB_ = −*D*_IT_, we need to reduce the H-parallax of the target by 1 H-pixel, to get *D*_HT_ = −*D*_HB_ − 1. We can do this by shifting the target either right by 1 H-pixel in the right monocular half-image or left in the left-image, −1 H-pixel. Figure 9 shows the same situation as Figure 7, but now the target dot in the left half-image has been shifted by −1 H-pixel. The I-parallax is now symmetrical, with a magnitude of |*D*_I_*|* = 3 I-pixels for both target and background dot (but the H-parallax is asymmetrical, with target *D*_HT_ = 1 H-pixel and background *D*_HB_ = −2 H-pixels).

In most stereo tasks and certainly in stereoacuity measurements, the relevant quantity is the relative disparity between target and background; small shifts in absolute disparity are irrelevant. Thus, the asymmetry of the H-parallax relative to the screen plane is not problematic. The critical point is that the symmetry of the I-parallax avoids possible monocular artifacts. In the Appendix, we present an algorithm for creating random-dot patterns containing a disparate target with a specified parallax relative to the background, so as to avoid cross-talk de-camouflage on column-interleaved displays.

**Figure 10:**
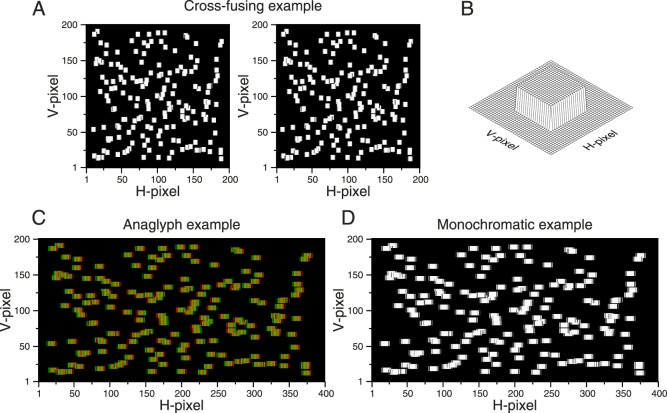
Example of a random-dot stereogram presented on a column-interleaved display with the artifact corrected. (A) Left and right images of the stereogram for cross-fusing. Background dots have a parallax of −2 H-pixels and the target dots have a parallax of 2 H-pixels (antialiasing was also used for reducing position artifacts). (B) Sketched version of the stereogram representing the 3-D percept. (C) Anaglyph version. Left and right images are drawn interleaved (the red filter should be placed in front of the right eye). Note that now the target dots are identical to the background dots. (D) Monochromatic version of panel C where the target cannot be discriminated. The dots in panels C and D are now rectangular, but the aspect ratio can be changed easily by increasing the height of the dot to produce a square dot on the screen.

Figure 10 shows an example random-dot stereogram, presented as if on a column-interleaved display, generated using this algorithm. The disparate target is still detectable in the anaglyph (Figure 10C) from the colored fringes. However, critically, it is not detectable in the merged binocular image (Figure 10D). Both target and background dots have the same width. This demonstrates that our algorithm successfully removes this artifact on column-interleaved displays.

**Figure 11:**
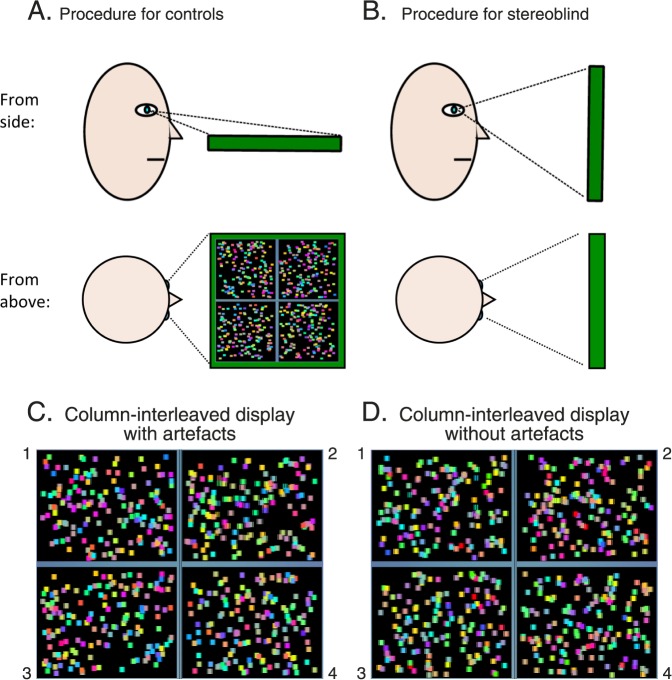
Testing-procedure examples of stimuli. (A) Sketch representing the testing procedure for controls. The tablet was horizontal so the cross talk was maximized and no 3-D was perceived. (B) Testing procedure for stereoblind participants. The tablet was held in the usual way, frontoparallel to the viewer. (C) Screen capture of one frame for the experiment with the column-interleaved artifact. The target is located in Quadrant 2 and is visible in this merged binocular image with careful viewing. (D) Screen capture of one frame for the experiment without the artifact. The target is located in Quadrant 1. The target is now totally camouflaged.

### Problem 4: Changes in viewing angle produce monocular motion artifacts

Even when the size of the target and background dots cannot be distinguished in the merged binocular image, there are other monocular artifacts that can still occur and make it possible to distinguish the left and right half-images. For example, we noted this issue previously in the case of anaglyph (Figure 4B), where the target can be identified from the colors of the fringes on either side of each dot. In a column-interleaved display, a similar problem occurs if the display is tilted about a vertical axis relative to the viewer, introducing motion parallax. Dots appear to jump horizontally, as left and right half-images become alternately visible to each eye. The direction of the jump is opposite for target and background dots. This can be observed in the Lang stereotest, for example, if the viewer tilts the card in his or her hand or turns his or her head from side to side. A careful observer could use this effect to identify the target even when viewing monocularly.

### Solution 4: Use dynamic stimuli to avoid monocular motion artifacts

The great advantage of digital displays over older autostereo systems is that they allow the use of dynamic stimuli, which effectively remove this cue. In dynamic stimuli, a new image is generated every frame, with the same disparity profile but a new pattern of random dots. If the refresh rate is rapid enough, then each dot will vanish before its monocular motion can be detected. The particular refresh rate required depends on the characteristics of the stimulus and display (e.g., dot size, tilt angle required for parallax inversion).

## Experimental verification

We wanted to verify empirically that the cross-talk de-camouflage artifact provides a visible monocular cue if parallax is applied incorrectly on a column-interleaved display, and at the same time confirm that our proposed solution eliminates the artifact. To this end, we asked two stereoblind observers and four controls to perform the stereoacuity task described under Methods. In Experiment 1, H-parallax was applied symmetrically, meaning that the target and background dots could be distinguished monocularly by their width when the tablet was held so that the two half-images were each visible to both eyes. In Experiment 2, I-parallax was applied symmetrically, with the intention of removing this dot-width artifact.

### Methods

#### Equipment

Experiments were performed using a NEO3DO tablet computer (http://www.neo3do.com/) running version 4.1.1 of the Android operating system on a 1.5 GHz dual-core Cortex A9 processor. The device has a diagonal screen size of 8.1 in. (17.3 cm wide × 10.9 cm high) and a resolution of 1280 × 800 pixels. It uses column-interleaved parallax-barrier stereoscopic 3-D as described previously. During experiments, the parallax barrier was activated and the viewing distance was 25 cm from the center of the screen.

#### Subjects

Six observers took part in both experiments. All observers were tested for visual spatial acuity (with the logMAR Uncrowded Test at 3 m) and stereoacuity (with the Randot stereo test). All participants had normal visual spatial acuity; two participants (001 and 002) had no stereovision (previously confirmed during orthoptic examination) and the rest had normal stereovision (Table 1). Participant 005 was one of the authors, but the other observers were unaware of the purpose of the experiments. All participants provided informed written consent. The study protocol was compliant with the Declaration of Helsinki and was approved by the Ethics Committee of the Newcastle University Faculty of Medical Sciences (approval number 00625).

**Table 1:**
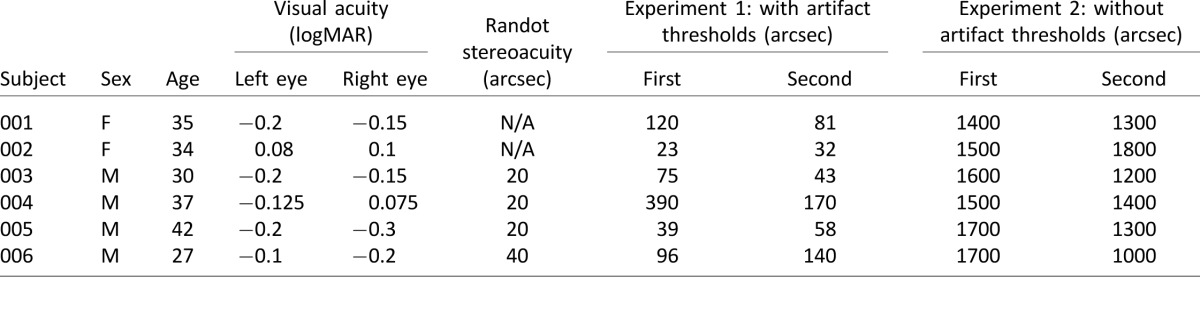
Results from Experiments 1 and 2.

#### Stereoacuity task

We used a detection stereoacuity task using a four-alternative forced-choice paradigm. The stimuli were four patches of dynamic and colored random dots (Figure 11), one containing a disparate target. The task was to identify the patch containing the target. The relative disparity between the target and background changed each trial using an adaptive Bayesian staircase (Treutwein, 1995). The characteristics of this staircase are described in detail elsewhere (Serrano-Pedraza et al., 2016); the threshold estimate was the value of the staircase after 30 trials. Each patch was 329 × 414 I-pixels (4.44 × 5.59 cm, horizontal × vertical) and made up of square dots 16 × 16 I-pixels. Antialiasing was used to position dots at noninteger pixels and to apply noninteger disparities (see Appendix).

Since in these experiments we were interested solely in the visibility of monocular cues, we instructed our control participants to view the tablet highly slanted about a horizontal axis (Figure 11A). This ensured that left and right half-images were highly visible to both eyes and destroyed any stereoscopic percept, effectively rendering these participants stereoblind. Our two stereoblind participants were allowed to hold the tablet normally (Figure 11B). Participants were not informed about which experiment they were performing. Each experiment was repeated twice and in a random order.

### Results

The results are presented in Table 1. In Experiment 1, with the dot-width artifact, all subjects performed as if they could see the target (mean disparity threshold <300 arcsec for all observers). Our two stereoblind subjects, who were holding the tablet normally, were also clearly able to detect this monocular cue, with one of them recording a stereo threshold of 23 arcsec. This confirms that this kind of cross-talk artifact is potentially a serious problem for column-interleaved displays, if the half-images are generated in the same way as for other stereoscopic displays.

In Experiment 2, we applied disparity in a way designed to remove the dot-width artifact. Now all subjects performed near chance. The staircase ended with thresholds of >1000 arcsec, which would classify the observers as stereoblind (recall that the observers with stereopsis were viewing the display in a way that removed stereo cues). This confirms that there were now no monocular cues which observers could exploit to produce an artificially low stereo threshold.

## Discussion

New technical advances are offering new ways of displaying 3-D content. The main objective of stereoscopic displays is to present different images to the left and right eyes in order to produce a vivid depth sensation. There is a wide range of methods that stereoscopic displays use to present each image to each eye; each method has advantages and drawbacks. For example, the field-sequential approach presents 2-D images to each eye interlaced temporally. This method can present images with high spatial resolution but introduces distinctive motion and depth artifacts (Hoffman, Karasev, & Banks, 2011). On the other hand, spatial interlaced methods (e.g., row-interlaced or column-interlaced) have better temporal resolution but lower spatial resolution (Johnson, Kim, & Banks, 2015).

Almost all current displays suffer from *cross talk*: when an image intended for one eye is partially or wholly visible to the other eye. Cross talk is often highly dependent on viewing angle, which makes it hard to correct for with software. Cross talk is always undesirable, because it reduces the depth percept. In tests of stereoacuity, cross talk is a particular problem because it can de-camouflage cyclopean objects which were intended to be detectable only via stereopsis, making it possible for subjects to “cheat,” consciously or otherwise, in these displays. However, these displays are often very convenient; for example, autostereo displays are particularly attractive for clinical use with children, given that no glasses are needed to perceive depth. So it is very desirable to think about how to design experiments so as to minimize these problems.

Row-interleaved and column-interleaved stereo methods at first sight appear symmetric—for example, row interleaving effectively turns square H-pixels into vertically elongated I-pixels; column interleaving turns square H-pixels into horizontally elongated I-pixels; row interleaving introduces vertical parallax; column interleaving introduces horizontal parallax. However, because horizontal parallax is what is relevant for stereopsis, these manipulations are not equivalent. The small vertical parallax introduced by row interleaving has no effect on stereopsis, and indeed is likely to be removed by a reflexive adjustment of vertical vergence. In contrast, the small horizontal parallax introduced by column interleaving interacts with the horizontal parallax applied as part of the stimulus. Thus, if you take the same half-images and display them either superimposed, row interleaved, or column interleaved, you can get different effects, even after accounting for the change in pixel aspect ratio.

One consequence is that stereograms which are robust to cross-talk de-camouflage when presented superimposed or row interleaved will present artifacts if their component half-images are presented on column-interleaved displays. This occurs because the I-parallax on the interleaved display will not be symmetric even if the H-parallax in the half-images is. In this article, we have shown that this artifact is visible and can be used to perform a stereoacuity task even when the observer is stereoblind or the display is viewed in a way that merges both images. In order to avoid these artifacts, stimuli need to be generated specifically for column-interleaved displays. We have explained how to do this and shown that this eliminates the artifact completely.

This does not mean that cross talk is no longer a problem. It is still undesirable, because it reduces stereoacuity. A viewer who is viewing an autostereo display from the wrong angle will achieve poorer stereo thresholds than he or she would have been capable of without cross talk. Thus, more sophisticated solutions which reduce cross talk—for example, dynamic parallax barriers which track and correct for viewer position—are highly desirable. Nevertheless, removing monocular artifacts is a key advance, for two reasons. First, clinically it may be more concerning if a stereoblind patient is wrongly categorized as having good stereoacuity than if a person with good stereoacuity scores below his or her true ability (the latter may trigger additional investigation which turns out to be unnecessary, but the former risks leaving a problem undetected). Second, observers will generally figure out what they need to do to perform the task. Thus, monocular artifacts are problematic precisely because stereoblind viewers are likely to learn if they can complete an otherwise impossible stereo task by tilting their head. However, by the same token, they will learn to avoid viewing the display from the wrong angle if this makes the task harder. Thus, although it would be good to remove cross talk altogether, removing its monocular artifacts is the most important component.

In summary, we have produced three “golden rules” for presenting cyclopean stimuli in a way that makes the task robust to monocular artifacts:

1. Use a task that is robust to parallax inversion
2. Apply parallax symmetrically to avoid cross-talk de-camouflage, taking particular care to achieve this in column-interleaved displays
3. Use dynamic stimuli to avoid monocular motion artifacts

If these rules are observed, we have shown that it is possible to run a stereoacuity task on a column-interleaved display that cannot be performed using monocular cues or artifacts. We hope that these recommendations will be useful to vision scientists interested in running stereo psychophysics experiments with these new display technologies.

## Supplementary Material



## Figures and Tables

**Figure 12:**
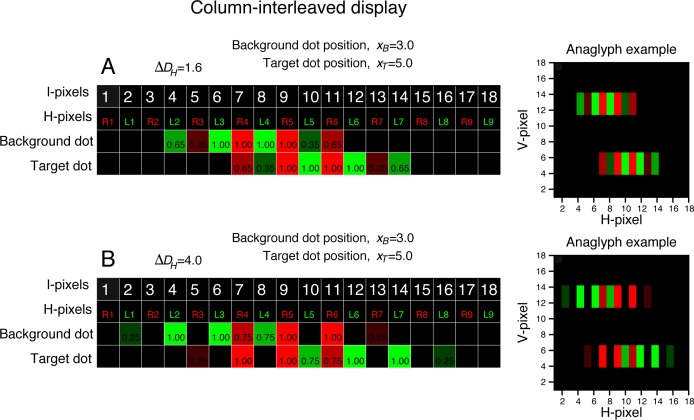
Avoiding position and monocular artifacts: examples with antialiasing. (A) Example showing a background dot and a target dot with relative parallax of Δ*D*_H_ = 1.6 H-pixels. The position of the background dot is *x*_B_ = 3 and the position of the target dot is *x*_T_ = 5. The final position of the target and background once the relative parallax is applied was calculated according to Equation 2. The dots are 4 H-pixels wide. The number inside the squares represents the digital driving level (see main text for details). (B) Same details as in panel A, but the relative parallax is Δ*D*_H_ = 4 H-pixels. The images on the right present the dots in an anaglyph version (the red filter should be placed in front of the right eye).

## Appendix: Algorithm for displaying a desired relative disparity without monocular artifacts on column-interleaved displays

This appendix explains in detail how to display a stimulus with a desired relative parallax (denoted Δ*D*_H_; in H-pixels) between the background and target in a way that avoids cross-talk de-camouflage on column-interleaved displays. This solution is for the case where the right half-image is presented in odd columns and left half-image in even columns. The same solution with minimal changes will work for displays with a different column arrangement (i.e., the odd columns are presented into the left eye). We use antialiasing to achieve subpixel disparities and positions.

For each dot, generate a random pixel position *x*, where *x* is a real (noninteger) number. If this position is assigned to the location *x*_T_ of the target, draw the dot at

in the left half-image and

in the right half-image.

Otherwise, if the position is assigned to the location *x*_B_ of the background dot, draw it at

in the left half-image and  (2)

in the right half-image (Equation 2).

This means that target dots have a parallax of

whereas background dots have a parallax of

Therefore, the relative parallax between target and background is

as required.

### Antialiasing method

In general, none of these numbers will be integers, since we use antialiasing to simulate dots at noninteger pixel values. To draw a dot which extends from *x*_1_ to *x*_2_, proceed as follows: Fill the H-pixel at *j* = floor(*x*_1_) with a digital driving level (i.e., luminance level) DDL = *rL*_F_ + (1 − *r*)*L*_D_, where *L*_D_ is the DDL of the dots (e.g., white), *L*_F_ is the DDL of the field on which they are presented (e.g., black), and *r*_1_ = *x*_1_ − floor(*x*_1_). For example, if the left side of a dot starts at position *x* = 2.3, this means that 30% of Pixel 2 should be filled and 70% covered by the dot. The following pixels should be filled with *L*_D_ until we reach Pixel *k*, where *k* is the smallest integer for which (*k* + 1) > *x*_2_. Pixel *k* is filled with DDL = (1 − *r*_2_)*L*_F_ + *r*_2_*L*_D_, where *r*_2_ = *x*_2_ − floor(*x*_2_).

Figure 12 shows two examples where the relative parallax between target and background is Δ*D*_H_ = 1.6 H-pixels (Figure 12A) and Δ*D*_H_ = 4 H-pixels (Figure 12B). For Figure 12A the position of the background dot is *x*_B_ = 3; according to Equation 2, the left-half side of the background dot starts at position *x*_BL_ = 2.35 (so Pixel L2 is 65% covered by the dot) and the right-half side starts at position *x*_BR_ = 3.65 (so Pixel R3 is 35% covered by the dot). The position of the target is *x*_T_ = 5; thus, according to Equation 2, the left-half side of the target dot starts at position *x*_TL_ = 4.65 (so Pixel L4 is 35% covered by the dot) and the right-half side starts at position *x*_TR_ = 4.35 (so Pixel R4 is 65% covered by the dot). The parallax for the target dot is then *x*_TL_ − *x*_TR_ = 0.3, and the parallax for the background dot is *x*_BR_ − *x*_BL_ = 1.3; thus, the relative parallax is Δ*D*_H_ = 1.3 + 0.3 = 1.6 H-pixels.

For Figure 12B, the relative parallax between target and background was Δ*D*_H_ = 4 H-pixels. The positions of the background and target dots are *x*_B_ = 3 and *x*_T_ = 5. According to Equation 2, the left-half side of the background dot starts at position *x*_BL_ = 1.75 (so Pixel L1 is 25% covered by the dot) and the right-half side starts at position *x*_BR_ = 4.25 (so Pixel R4 is 75% covered by the dot). The left-half side of the target dot starts at position *x*_TL_ = 5.25 (so Pixel L5 is 75% covered by the dot) and the right-half side starts at position *x*_TR_ = 3.75 (so Pixel R3 is 25% covered by the dot). The parallax for the target dot is then *x*_TL_ − *x*_TR_ = 1.5, and the parallax for the background dot is *x*_BR_ − *x*_BL_ = 2.5; thus, the relative parallax is Δ*D*_H_ = 2.5 + 1.5 = 4 H-pixels. Note that in both examples, the H-parallax is not applied symmetrically to the target and background dots, but the relative parallax is as desired.

This procedure makes it impossible to detect the target in the merged binocular image. This is demonstrated in Figure 10, which shows a random-dot pattern drawn using the algorithm of Equation 2. Figure 10A shows an example of a random-dot stereogram. The same random-dot example is shown in a merged binocular image (Figure 10B) as it would appear on a column-interleaved display, using anaglyph to distinguish the left and right images. Figure 10C shows a monochromatic version of the same pattern; the target is now fully camouflaged.
