# Supplementary figures and images for: Avoiding monocular artifacts in clinical stereotests presented on column-interleaved digital stereoscopic displays
Source: J Vis. 2016 Nov 16;16(14):13. doi: 10.1167/16.14.13 (PMC5114011; doi:10.1167/16.14.13)

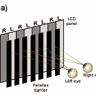

Supplement: Supplementary file 1 [file JOV-05438-2016-s01-ICON.gif]
